# Cerebral Proliferative Angiopathy in a Child, Five Years after an Intraventricular Hemorrhage and Negative Catheter Angiography

**DOI:** 10.5334/jbsr.3247

**Published:** 2023-08-03

**Authors:** Uri Singfer, Edward Baert, Luc Defreyne

**Affiliations:** 1 Department of Vascular and Interventional Radiology, Ghent University Hospital, BE; 2 Department of Neurosurgery, Ghent University Hospital, BE

**Keywords:** neuroradiology, cerebrovascular diseases, hemorrhagic stroke, pediatric/children, arteriovenous malformations

## Abstract

Cerebral proliferative angiopathy (CPA) is a rare cerebrovascular disease comprised of a diffuse network of blood vessels, often spread over several lobes. Due to the presence of normal brain tissue within the malformation, complete resection is not desirable. Several imaging characteristics help to distinguish CPA from brain arteriovenous malformations. We report the case of a patient diagnosed with CPA five years after a spontaneous intraventricular hemorrhage and an initial negative angiogram.

**Teaching Point:**

The radiologist should be able to differentiate CPA from brain AVM to prevent potentially harmful treatment.

## Introduction

Brain arteriovenous malformations (bAVMs) are phenotypically extremely heterogenous lesions which may have a compact or diffuse nidus. In cases of diffuse nidi, cerebral proliferative angiopathy must be included in the differential diagnosis and ruled out before treatment.

## Case History

A 16-month-old female was referred to an emergency department in a university hospital after a generalized seizure. A nonenhanced computed tomography (CT) of the brain revealed a large intraventricular hemorrhage, mostly into the right lateral ventricle. An urgent brain magnetic resonance imaging (MRI) showed no vascular pathologies. Craniotomy was performed the next day and the choroid plexus of the right lateral ventricle, which was identified as the cause of the bleeding, was clipped. Postoperative MRI and digital subtraction angiography (DSA) could not show any shunt-lesions. The next five years were uneventful. The contact between the patient and the hospital was unfortunately lost and the patient did not undergo further investigations.

**Figure 1:**
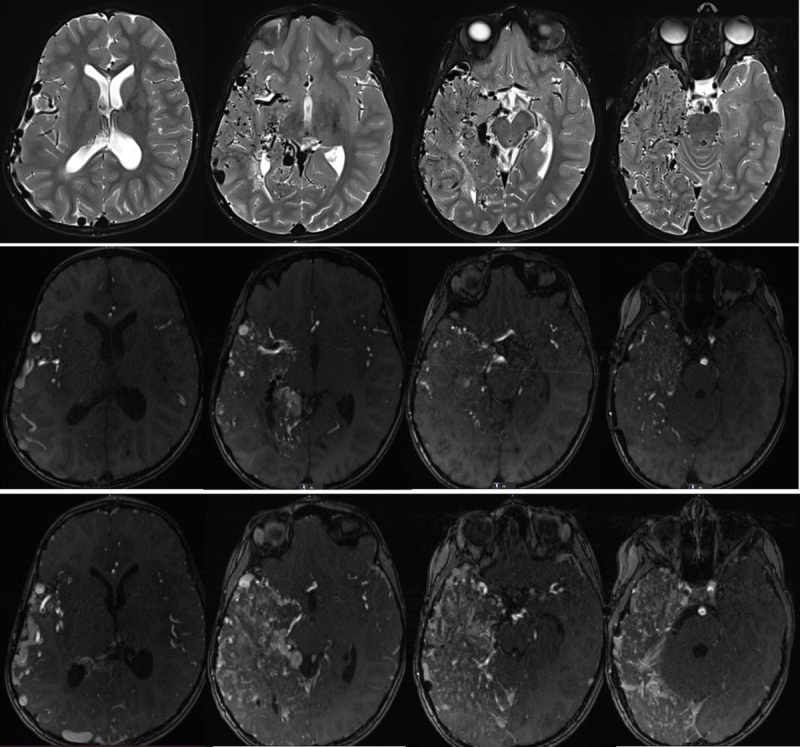
Axial T2WI (upper row), axial TOF (middle row), and axial TOF after IV-gadolinium (lowest row) show a diffuse vascular network in the right hemisphere within normal brain parenchyma.

**Figure 2:**
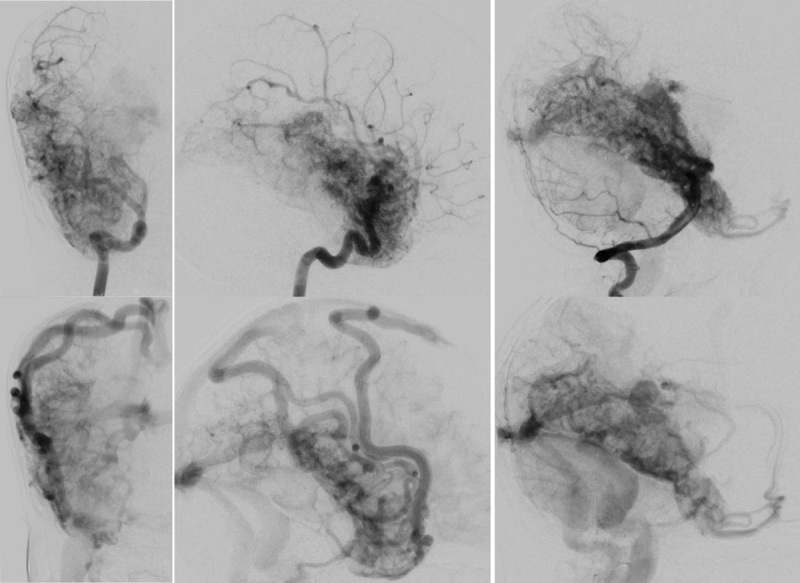
Right internal carotid artery digital subtraction angiography (DSA) in frontal (left images) and lateral (middle) views and right vertebral artery DSA in lateral views (right images) during the arterial (upper row) and late arterial (lower row) phases show a diffuse vascular network in the right hemisphere with no dominant arterial feeders and persistent opacification into the late arterial phase. Venous drainage is to the superior sagittal sinus and deep venous system.

Five years later the patient was admitted to the same emergency department with acute right retro-orbital headache, diplopia, and nausea. CT excluded hemorrhage but revealed multiple enlarged vessels throughout the right hemisphere. A subsequent MRI revealed a diffuse vascular network supplied by branches of the right middle and posterior cerebral arteries and drained by enlarged veins to the superior sagittal sinus and internal cerebral vein (Figure 1). On DSA, there were no dominant arterial feeders, no related aneurysms nor proximal arterial stenoses. Persistent opacification of the malformation in the late arterial phase and some early venous filling was noted (Figure 2). The diagnosis of cerebral proliferative angiopathy was made.

**Figure 3:**
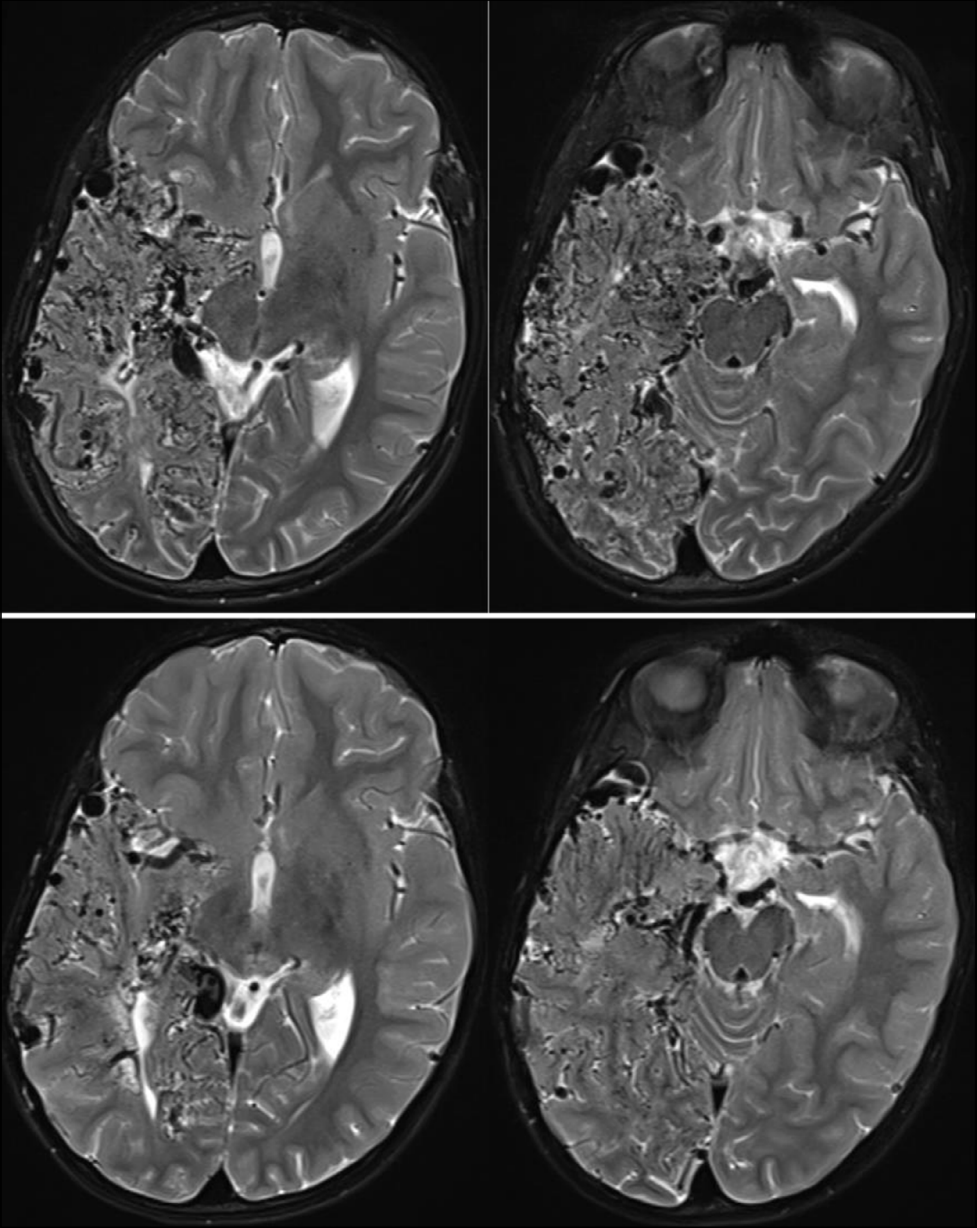
Axial T2WI during second presentation (lower row) and five years later (upper row) demonstrate progression of the vascular network in the right temporal and occipital lobes.

Three days after admission, symptoms were resolved, and the patient was discharged home the same week. During the following six years she experienced seven more episodes of right retro-orbital headache, diplopia, and nausea. In one episode she presented with left hemiparesis. The symptoms lasted for about 24–48 hours each time and resolved spontaneously. On MRI, which was performed in each episode, the malformation showed slow progression with no evidence of hemorrhage or infarction (Figure 3). Because of the relatively stable clinical status and the extent of the malformation, conservative treatment was elected.

## Comments

Cerebral proliferative angiopathy (CPA) was first described in 2008 by Lasjaunias et al. based on a series of 49 patients whom the authors identified as harboring a diffuse vascular lesion which differs from the ‘classical’ bAVM in morphology, clinical manifestation and natural history [1].

On cross-sectional imaging, CPA can be recognized as a diffuse network of blood vessels within identifiable normal brain parenchyma, without dominant arterial feeders. The lesion often extends to the thalamus and basal ganglia and is usually spread over several lobes. On DSA there should be persistence of contrast in the malformation into the late arterial or early venous phase, a near-normal arteriovenous transit time and normal to slightly dilated draining veins. Stenosis of proximal arteries and transdural blood supply can often be seen in CPA patients and are a sign of angiogenesis. Flow-related aneurysms usually do not occur [1]. Clinically, CPA may manifest with seizures, headaches, and stroke-like symptoms. Importantly, the risk of hemorrhage seems relatively low in patients with CPA, and it was present in 12% of patients in the aforementioned case series [1].

The presumed pathomechanism in CPA involves uncontrolled angiogenesis, possibly due to chronic perilesional hypoperfusion, leading to the formation of a diffuse arterial network, often in several lobes [1]. This theory is supported by several studies that, using perfusion weighted imaging, positron emission tomography (PET) or Single-photon emission computed tomography (SPECT), demonstrated hypoperfusion of the affected hemisphere in CPA patients [2]. To address the problem of hypoperfusion, several groups utilized indirect revascularization techniques such as calvarial burr holes and encephaloduroarteriosynangiosis (EDAS) and reported favorable outcomes [2]. Conversely, surgical resection, radiosurgery or large nontargeted embolization of the malformation should be avoided as they carry a high risk of permanent neurological deficit due to damage to the interspersed normal neural tissue [1].

Among reported CPA patients, our patient had an unusual medical history that included intraventricular hemorrhage, neurosurgery and a negative angiogram, five years before the CPA diagnosis. This case, to our knowledge, is the first documentation of CPA development after a negative DSA. It supports the hypothesis that CPA involves formation of new blood vessels.

## Conclusion

CPA has several unique radiographic characteristics distinguishing it from bAVMs. This case documents the progressive nature of CPA.
